# How positive and negative feedback following real interactions changes subsequent sender ratings

**DOI:** 10.1038/s41598-025-91750-1

**Published:** 2025-03-03

**Authors:** Antje Peters, Jendrik Witte, Hanne Helming, Robert Moeck, Thomas Straube, Sebastian Schindler

**Affiliations:** 1 Institute of Medical Psychology and Systems Neuroscience, University of Münster, Von-Esmarch Straße 52, 48149 Münster, Germany; 2 Otto Creutzfeldt Center for Cognitive and Behavioral Neuroscience, University of Münster, Münster, Germany

**Keywords:** EEG/ERPs, Social feedback, Social influence, Evaluative feedback, Other-view updating, Expectation, Cognitive neuroscience, Emotion, Social neuroscience, Visual system, Human behaviour

## Abstract

Social evaluative feedback informs us about how others perceive us, constantly updates our expectations of what to receive, and simultaneously changes our view of the sender. However, little is known about the neuronal and behavioral responses when receiving incongruent positive or negative social evaluative feedback. This study (*N* = 40) investigated how receiving feedback from peers after a real-life interaction modulates behavioral responses and Event-Related Potentials (ERPs). Specifically, ERP modulations by feedback being incongruent with the self-view and incongruent with the feedback expectation were examined along the whole processing stream. Feedback was manipulated such that one peer provided overly positive feedback and the other overly negative feedback, with random computer feedback as a control condition. Behaviorally, participants updated their feedback expectations according to the feedback received from the ‘negative’ and ‘positive’ peers and rapidly changed ratings of the sender towards their positive or negative behavior. Concerning ERPs, separate effects of feedback incongruence based on the feedback expectation or self-view were found during the mid-latency processing stages. Subsequently, both types of incongruence increased late ERP amplitudes, which were also increased when participants substantially changed the ratings of the peer senders. This is the first study that combined neuronal and behavioral measures of evaluative feedback processing, emphasizing that incongruent feedback elicits mid-latency modulations and subsequent updating processes associated with increased late amplitudes. In addition, we find rapid behavioral changes in the ratings for the senders based on their feedback behavior.

## Introduction

Social evaluative feedback is part of our daily social interaction. Positive and social evaluative feedback appears vital to fulfilling the psychological need to belong, which has been crucial to survival in the past^1^. On the other side, negative feedback and not being socially embedded increases the risk of depression, anxiety, and premature death^2^. However, other’s social evaluative feedback may or may not agree with the self-view. Deviant feedback creates tension with our striving for self-congruence in the self-view^3^, which requires updating the self-view or the other-view to eliminate discrepancies. Social media amplifies this dynamic. Positive feedback can enhance social sharing and strengthen social bonds in social media^4^. Social media platforms provide constant feedback loops, where likes, comments, and shares serve as immediate indicators of approval or disapproval^5^. This instant feedback can influence self-perception, sometimes leading to increased confidence but also to comparison and self-doubt. The visibility and scale of feedback on social media have intensified the influence of social feedback, making it both a powerful tool for connection and self-expression but also a potential source of stress or pressure in everyday life that can even influence neurodevelopment^6^.

So far, neuroscientific studies have largely focused on how feedback-senders influence social evaluative feedback processing, identifying relevant characteristics such as expertise^7^ or attributed social source of the feedback^8^. However, the feedback sent informs about the sender, changing the attributed sender characteristics. Senders providing overly negative feedback will be updated accordingly regarding what we expect from them and how we think about the sender. Nevertheless, no study has examined how the sender’s behavior affects the feedback-related neuronal responses and updating of the other-view and the feedback expectations.

The changes in other-related updating processes are yet unclear. Some behavioral findings suggest that receiving positive social evaluative feedback enhances the interest in the feedback sender and the likelihood of reciprocal positive behavior^9^ or feedback^10^. Negative behavior has been shown to elicit punishment in dilemma games^11,12^. Negative social evaluative feedback from others also induces negative assumptions about the sender^13^, reduces sender attractiveness^14^, and the likelihood of reciprocal negative behavior^15^ or feedback^16^.

Concerning feedback expectations, findings show that people view themselves as more positive than others and have positively biased feedback expectations^17,18^. A recent model for predictive processing by Kube and colleagues^19^ proposes that healthy people receiving disconfirming information should selectively update their expectations. Having negative expectations but receiving disconfirming positive feedback leads to positive information generalization and expectation updating. After disconfirming negative information, cognitive immunization maintains the original positive expectation^19–21^.

In studies examining visual social evaluative feedback stimuli, a recent review showed that the examined ERPs toward feedback span from the early P1/N170 components to the Late Positive Potential (LPP)^8^. Studies on social evaluative feedback processing often refer to an N1^8^ component, while feedback studies more commonly refer to this component as the N170^22,23^. In this paper, we will follow this convention. Based on studies using visual stimuli in general, the suggested functions and related cognitive processes of these ERPs have been described, which can be roughly divided into early, mid-latency, and late ERPs. Early ERPs are suggested to show higher automaticity in responding^24,25^, while later components or specific interactions might predominantly depend on higher-order processes (e.g., more controlled evaluation processes)^26,27^. These early responses to social evaluative feedback stimuli are found in the P1 and N170 range (~ 80 to 100 ms and ~ 120 to 170 ms post feedback) over occipital sensors^8,28,29^. The P1 and N170 are both strongly influenced by sensory strength^30–32^ and other low-level visual information^33–35^. P1 and N170 amplitude increases are related to enhanced early visual processing, such as typically found in studies manipulating attention^36,37^. The function of P1 has sometimes been related to the inhibition of irrelevant information^38,39^, with mixed results^40^, while the N170 component has been reliably related to the amplified processing of stimuli^30,38^. Increases of the P1 or N170 amplitudes can be observed in some social evaluative studies, but these seem to reflect tonic differences in feedback expectations^28^ or feedback decisions^29^, meaning that whenever participants are prepared that they will receive important feedback (such as the block-wise instruction about relevant vs. irrelevant senders).

Concerning mid-latency ERPs, the Early Posterior Negativity (EPN) is observed as differential occipital-temporal negativity when contrasting emotional and neutral stimuli and is typically observed between 200 and 300 ms^45,46^. The EPN is related to early emotional tagging and attention processes toward relevant information^47–49^. Based on findings showing a vulnerability to specific competing tasks, the EPN has been described as a bottleneck in further emotional differentiation^50,51^. EPN amplitudes are typically increased for socially relevant vs irrelevant feedback (e.g., ‘human’ vs. ‘computer’ feedback) and emotional compared to neutral evaluative feedback^8,52–55^. In the roughly same time window, the Feedback-Related Negativity is observed over frontal sensors, typically scored as a relative negativity for unexpected and/or negative feedback^56,57^. In social evaluative feedback studies, more typically, unexpected feedback and, in some studies, unexpected negative feedback increased the FRN amplitude^8^.

Among late ERPs, the P3 is divided into different components, typically at least into an earlier P3a and a later P3b component, the former with a frontal and the latter with a parietal distribution. While the P3a relates to stimulus novelty, the P3b relates to subsequent and elaborate stimulus processing and updating^58,59^. The Late Positive Potential (LPP) is part of the family of late positivities, emerging from approximately 400 ms onwards up to seconds after stimulus appearance. It is identified by contrasting emotional and neutral stimuli and indicates stimulus evaluation and controlled attention processes. While topographies often show a centro-parietal distribution, these vary depending on stimulus types and tasks^24,60^ and are hypothesized to reflect the activation of broad and distributed brain regions^61,62^. The LPP is related to elaborate stimulus processing, including stimulus evaluation and emotional appraisal, self-referential processing, and information integration^27,63^. Findings on social evaluative feedback show that more relevant (e.g., social vs. non-social) senders reliably increase the LPP^8^. Concerning feedback valence (i.e., positive vs. negative social evaluative feedback), valence differences are rarely observed, while, like for the EPN, LPP responses for emotional evaluative feedback are larger than for neutral feedback^8,52–55^.

This study investigated how social behavior affects feedback processing and behavioral updating of the sender and the feedback expectation. We realized a social interaction of three participants each, after which EEG was measured. Participants received supposedly social feedback from the two interaction partners. Feedback was manipulated according to the self-view, with one peer providing overly negative feedback and another providing overly positive feedback. We predicted participants would update their expectations according to the feedback valence, and, importantly, we also expected that sender behavior would influence the updating of the trait adjectives. We tested how social evaluative feedback being incongruent with the self-view, or with the feedback expectation, would affect the whole sequence of ERPs effects of early (P1, N170), mid-latency (EPN, FRN), and late (LPP) ERPs. Importantly, we hypothesized that after participants updated their sender ratings, those trials would be associated with increased LPP amplitudes.

## Method

### Participants

We determined the sample size based on the most similar previous study that examined ERPs for 40 participants for feedback in real dyadic interactions^64^. A sample of 41 healthy participants was recruited in Münster through the student newsletter (ASTA) and personal networks. One participant aborted testing, leading to the final sample of forty native-level German-speaking participants (26 females, 13 males, 1 diverse) with a mean age of 23.88 years (*SD* = 2.69). All participants were right-handed, had normal or corrected-to-normal vision, and reported no previous or current neurological or psychiatric disorders. All participants provided written informed consent and received 12 Euros per hour of participation or course credit. The study was approved by the Deutsche Gesellschaft für Psychologie ethics committee (2021-10-17WV). We confirm that all experiments were performed in accordance with relevant guidelines and regulations at the University of Münster.

### Material and stimuli

We assigned and matched 180 adjectives to four word lists (see Table 1). Adjectives were rated using the self-assessment manikins^65^ for valence, arousal, concreteness, and self-relevance of personality evaluations in a separate sample of student participants. Linguistic properties were matched using the dlex database^66^. The list assignment to the senders was counterbalanced across participants. Participants were measured with the Beck-Depression-Inventory^67^, the Rosenberg self-esteem scale^68^, the Fear of Negative Evaluation Scale^69,70^, the Anxiety Sensitivity Scale^71^, and the Social Phobia Scale^72^, which were assessed as part of a bigger research project.

**Table 1:** Comparison of the four word lists.

Variable	List 1 (N = 45)	List 2 (N = 45)	List 3 (N = 45)	List 4 (N = 45)	*F*-value (3,176)	*p*-Value
Valence	5.21 (2.26)	5.24 (2.33)	5.35 (2.36)	5.43 (2.29)	0.08	0.969
Arousal	3.99 (0.66)	4.07 (0.74)	3.93 (0.78)	4.13 (0.71)	0.71	0.550
Self-relevance	5.95 (0.91)	6.09 (1.00)	6.15 (0.98)	6.24 (0.90)	0.13	0.942
Concreteness	5.51 (1.11)	5.51 (1.14)	5.48 (1.23)	5.62 (1.24)	0.71	0.551
Word length	9.91 (2.00)	9.96 (1.87)	9.96 (1.71)	9.71 (1.84)	0.18	0.912
Word frequency	270 (368)	262 (307)	255 (348)	247 (363)	0.04	0.991
Regularity	107 (240)	107 (173)	101 (208)	135 (255)	0.23	0.877

Standard deviations appear in parentheses below the means. Valence = 1 highly negative, 5 = neutral, 9 = highly positive; Arousal 1 = very low, 9 = very high; Concreteness 1 = very concrete, 9 = very abstract. Word frequency is depicted per million.

### Procedure

**Fig. 1:**
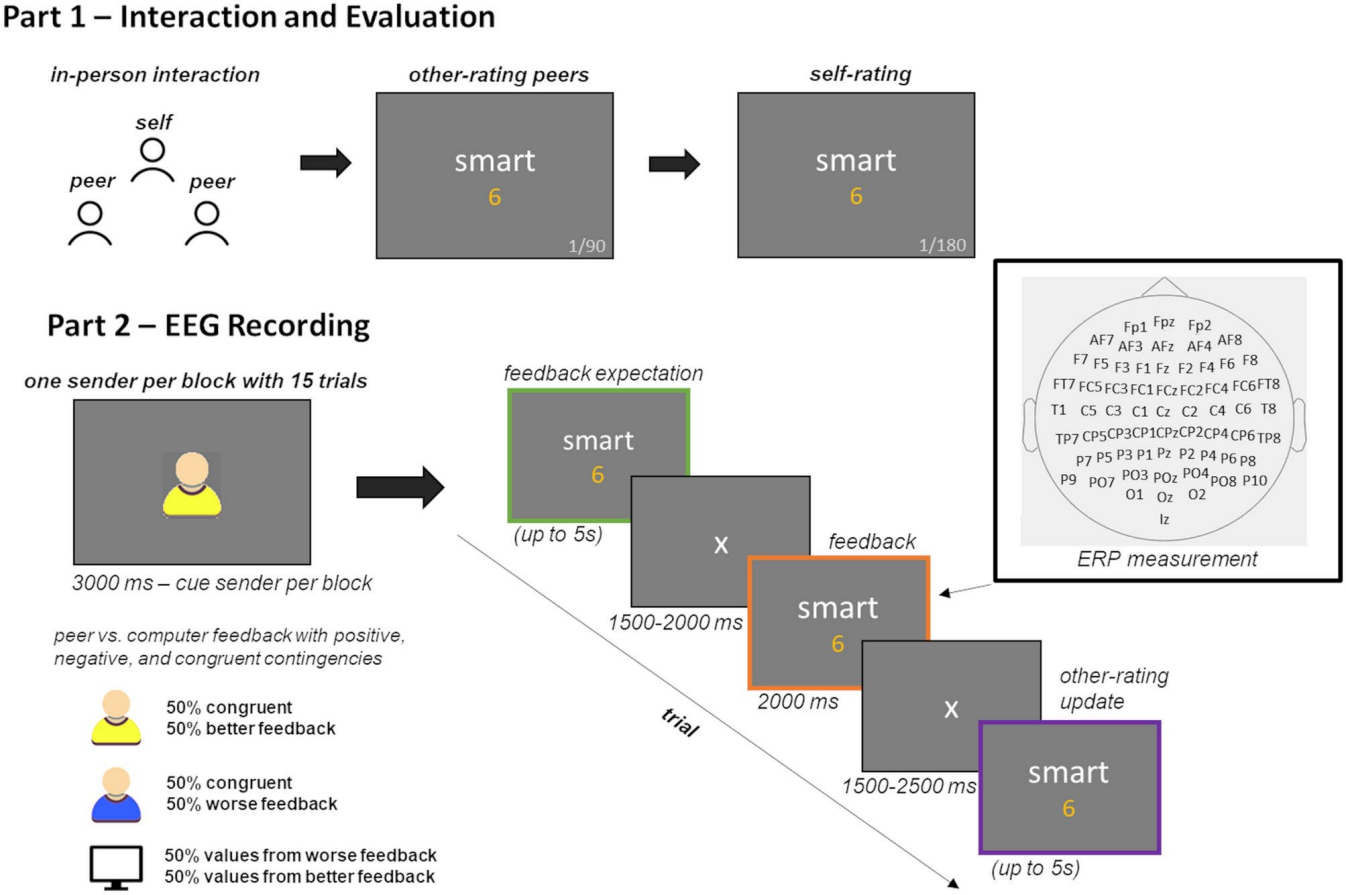
Experimental Setup. (**Part 1**) Group conversation of three formerly unknown peers following a structured interview. One of the three was a confederate who was only present for credibility reasons. Each of the two participants evaluated their fellow peers on 90 adjectives and themselves on all 180 adjectives on a scale ranging from 1 to 9. (**Part 2**) While recording EEG data, participants received manipulated feedback from their two peers and computer feedback. A ‘positive peer’ provided more positive feedback (50% congruent, 50% better), a ‘negative peer’ provided more negative feedback (50% congruent, 50% worse), while the computer feedback provided random feedback with the peer-feedback values on different traits. Each trial started with presenting the adjective and asked participants about their expectations regarding the feedback. After feedback was presented, participants re-evaluated the peer on this trait adjective.

The study took place at the Institute of Medical Psychology and Systems Neuroscience (IMPS), where a confederate and two participants who did not know themselves before interacted (see Fig. 1). After giving informed consent, the study started with a group conversation using structured interaction protocols that asked participants about their personality, strengths, and weaknesses^29,54,64^.

After the interaction, participants were prepared in two different EEG rooms for measurement and rated the other two interaction partners on separate trait adjectives. For each peer, two lists (see Table 1) were selected, and each peer was rated on 90 unique trait adjectives on a scale ranging from 1 (not applicable) to 9 (highly applicable). Word lists per peer sender valence were counterbalanced across participants. Then, they rated themselves on the complete set of all 180 adjectives and filled in the questionnaires (see Fig. 1). Based on these self-ratings, the subsequent feedback from the ‘positive’ and ‘negative’ peers was manipulated. In the subsequent main EEG experiment, participants were instructed to receive feedback from the other two participants and random computer feedback as a control condition. Each sender was introduced with alternating blocks of fifteen trials, where each block was cued with an icon (human yellow or blue, counterbalanced, and a computer icon; see Fig. 1) and the respective peer surname. In each trial, participants were first asked for their expectations on each trial, i.e., to rate how they believed the respective sender evaluated them (same scale from 1 to 9) and had up to 5 s to decide. Subsequently, they were shown the supposedly given feedback, and for feedback onset, ERPs were calculated (see Fig. 1). Then, participants were asked to re-evaluate the respective peer sender, again self-paced but restricted to 5 s. No trait update was requested for computer feedback. The displayed feedback was manipulated based on the participant’s self-evaluation. Each peer provided feedback on the 90 trait adjectives on which s/he had been rated before. One peer provided overly positive feedback (‘positive peer’; 90 trials, 50% congruent, 50% more positive than the self-rating of the participant). The other peer provided overly negative feedback on the 90 trait adjectives on which s/he had been rated before (‘negative peer’; 90 trials, 50% congruent, 50% more negative than the self-rating of the participant). Positive and negative feedback trials deviated equally distributed by 1 to 3 points from the participant’s initial self-evaluation. The feedback ratios and valence deviations were chosen according to a previous study using real interactive behaviour^64^ to ensure the credibility of the feedback provided. The computer feedback used the feedback values from the two peers but randomly assigned to valence-matched adjectives. The experiment was programmed and run with Matlab (Version R2019b; Mathworks Inc., Natick, MA; http://www.mathworks.com) using the Psychophysics Toolbox (Version 3.0.15)^38,39^. Following the experiment, participants were debriefed.

### EEG recording and preprocessing

EEG data were recorded from 64 BioSemi active electrodes using BioSemi’s Actiview software (version 8.11; www.biosemi.com), according to the 10/20 layout (cf. Fig. 1). Electrode offsets were kept below ± 40 microvolt, as recommended, as an indicator of electrode noise. Additionally, four external electrodes measured horizontal and vertical eye movements. A Common Mode Sense active electrode (CMS) and a Driven Right Leg passive electrode (DLR) were used as ground electrodes. The offline data was preprocessed with BESA software (version 6.0; www.besa.de). The data was referenced to the average reference and filtered with a 0.1 Hz high-pass forward filter (6 dB/oct) and a 40 Hz low-pass zero-phase filter (24 dB/oct). A predefined source model was applied to the data, combining three topographies accounting for EOG activities, consisting of horizontal and vertical eye-movement and blinks (HEOG, VEOG, blink) with 12 regional sources modeling the different brain regions. The adaptive artifact correction method then performed a principal component analysis (PCA) for segments where the correlation between data and artifact topography exceeded the HEOG (150 µV) or VEOG (250 µV) thresholds. All PCA components explaining more than the minimum variance were maintained, and then recorded data was decomposed using all topographies into a linear combination of brain and artifact activities^75^. The remaining artifacts were rejected based on an absolute threshold (< 120 µV), signal gradient (< 75 µV/∂T), and low signal (i.e., the *SD* of the gradient, > 0.01 µV/∂T). Noisy EEG sensors were interpolated using a spline interpolation procedure. The filtered data was segmented in epochs from 200 ms before feedback onset to 1500 ms after stimulus presentation with a baseline correction from 100 ms before the stimulus. For ERP analyses, we examined mean amplitudes of the P1 (80–100 ms), N170 (130–170 ms), EPN (270–370 ms), FRN (210–310 ms), and LPP components (400–1000 ms). We identified and averaged the P1, N170, and EPN over symmetrical occipito-temporal sensors (six electrodes: PO7, P7, P9, PO8, P8, P10), the FRN over a frontocentral cluster (two electrodes: FCz, Fz), and the LPP over a broad central cluster (twenty-five electrodes: F3, F1, Fz, F2, F4, FC3, FC1, FCz, FC2, FC4, C3, C1, Cz, C2, C4, CP3, CP1, CPz, CP2, CP4, P3, P1, Pz, P2, P4). For statistical analyses of ERP data, see the paragraph below.

### Statistical analysis

Statistical analyses were performed using JASP (https://jasp-stats.org/)^76^. For behavioral effects, we tested other updating and feedback expectation ratings. Other updates were calculated based on differences between the initial rating and the re-evaluation after receiving the social evaluative feedback, where negative values index negative and positive values are positive changes, averaged across each block. A Repeated Measures ANOVA with the factors sender (two levels: ‘positive peer’ vs. ‘negative peer’) and block order (six levels; block 1 to block 6) was performed. Concerning feedback expectation, for adjectives with a negative valence, the rating r was inverted (r’ = 10 − r), so high values correspond to high positive ratings. Average expectation ratings were calculated for each of the six blocks containing 15 trials per sender. A Repeated Measures ANOVA was performed with the factors sender (three levels: ‘positive peer’, ‘negative peer’, ‘computer’) and block order (6 levels: block 1 to block 6). To test for the earliest differences, behavioral analyses were additionally performed in the first block (15 levels: trial 1 to trial 15). Concerning ERPs, we explored ERP differences among the peers for their congruent and incongruent feedback based both on their self-view and feedback expectations. Here, we performed explorative analysis within the peer sender and the different feedback types based on the self-view or based on the feedback expectations. For self-view congruence, we ran two by two repeated Measure ANOVAs with the factors sender (two levels: ‘positive peer’ vs. ‘negative peer’) and feedback type (two levels: self-congruent vs. self-incongruent positive or negative feedback). For feedback expectations, we ran two by three repeated Measure ANOVAs with the factors sender (two levels: ‘positive peer’ vs. ‘negative peer’) and feedback type (three levels: worse than expected, congruent, and better than expected). While self-view violations were manipulated to obtain equal trial numbers concerning feedback expectations, these numbers differed between categories (see Table 2), with some conditions containing low trial numbers, specifically for worse than expected feedback from the ‘positive peer’.

**Table 2:** Trial numbers used for ERP analyses.

	‘Negative peer’	‘Positive peer’
Self-view		
Self-incongruent (negative or positive)	40.30 (4.30)	40.08 (3.83)
Self-congruent	40.33 (5.15)	40.33 (3.96)
Feedback-expectation		
Worse	30.85 (9.03)	8.87 (4.78)
Congruent	16.80 (5.01)	20.88 (8.29)
Better	24.85 (9.10)	44.44 (12.02)

Standard deviations are displayed in parentheses beyond means.

Finally, trials were categorized according to the subsequent updating of the peers, with ERP analyses for the degree of updating by three levels: updating 0, updating 1, updating > 1), again with differences between trial numbers (updating 0 M = 53.97, *SD* = 13.32; updating 1 M = 56.33, *SD* = 11.15; updating > 1 M = 44.55, *SD* = 13.94). For completeness, we tested for ERP differences between all trials from peer and computer senders using repeated Measures ANOVAs (see Supplementary Section 1). For Repeated Measure ANOVAs, *p*-values and effect sizes were corrected according to Greenhouse–Geisser whenever the Mauchly test violated the assumption of sphericity, marked in the results section (*), while uncorrected degrees of freedom were reported. Post-hoc comparisons used the Bonferroni-Holm procedure. For block order, we used Helmert contrasts to compare the linear trends of each block against all following blocks. Partial eta-squared (η_P_^2^) and Cohen’s d were estimated to describe effect sizes^77^.

## Results

### Behavioral data

#### Other rating update

**Fig. 2:**
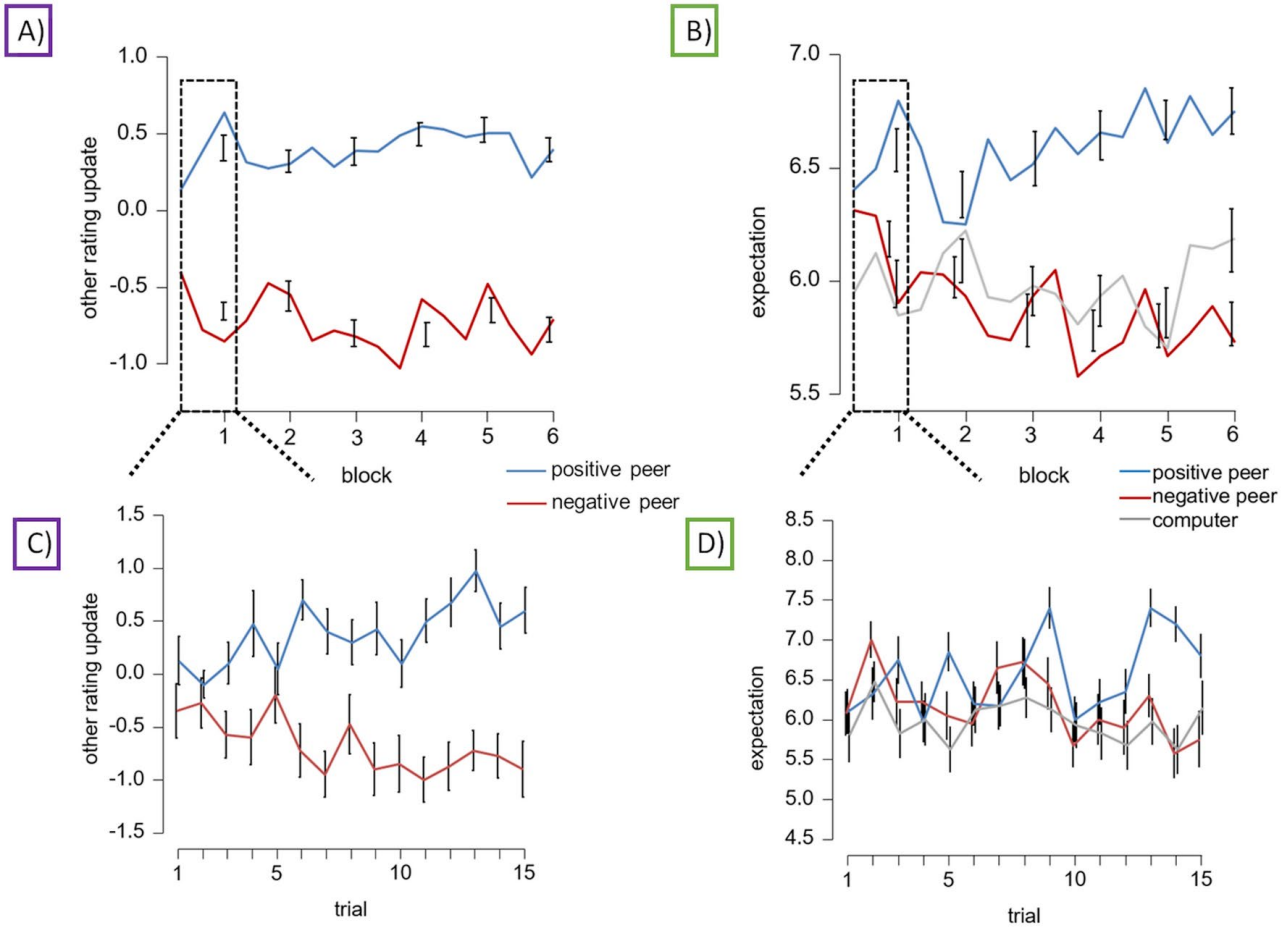
Other rating update and feedback expectation (**A**) Other-related updating after receiving feedback over time. Other updating values range from negative to positive, where the negative values index changes in the negative direction and vice versa for positive values. (**B**) Feedback expectation over time for all senders. Rating values range from 1 (most negative) to 9 (most positive). Note: For adjectives with a negative valence, the rating r was inverted (r’ = 10 − r), so high values correspond to high positive ratings. In A and B, averages of five trials each have been depicted to enable a closer look at the time course (solid lines), and analyses were performed across the mean of all six blocks (error bars). (**C**) and (**D**) highlight changes in ratings (**C**) and expectations (**D**) for the first 15 trials of the first block.

We tracked the progression of the other re-evaluation over time for the two peer senders (see Fig. 2A). The ANOVA revealed a statistically significant difference for the factor sender (*F*_(1,39)_ = 153.23, *p* < 0.001, η_P_^2^ = 0.770), no main effect of the block (*F*_(5,195)_ = 1.14, *p* = 0.347, η_P_^2^ = 0.028), and an interaction between the sender and block (*F*_(5,195)_ = 2.83, *p* = 0.017, η_P_^2^ = 0.068). Post-hoc comparisons found that ratings were significantly more positive for the ‘positive peer’ than for the ‘negative peer’ (*t*_(39)_ = 11.63, *p*_holm_ < 0.001, Cohen’s d = 1.751). Concerning the interaction, while in each block, the ‘positive peer’ was updated more positively than the ‘negative peer’ (*ts* > 7.04, *ps* < 0.001, Cohen’s ds > 1.364), we observed significant linear trends for the first (*F*_(1,39)_ = 4.26, *p* = 0.046, η_P_^2^ = 0.099) and second block (*F*_(1,39)_ = 13.25, *p* < 0.001, η_P_^2^ = 0.254). This was based on increasing differences in positive and negative ratings for the first two blocks compared to the subsequent blocks. From the third block onwards, no further significant trends were observed (*Fs* < 1.86, *ps* > 0.181).

A more detailed look at the first block for the first 15 trials showed a significant difference for the factor sender (*F*_(1,39)_ = 135.18, *p* < 0.001, η_P_^2^ = 0.776, see Fig. 2C), no main effect of the trial (*F*_(14,546)_ = 0.60, *p* = 0.864, η_P_^2^ = 0.015), and an interaction between the sender and trial (*F*_(14,546)_ = 2.15, *p* = 0.009, η_P_^2^ = 0.052). This interaction concerning re-ratings was best explained by a linear contrast of increasing differences (*F*_(1,39)_ = 19.13, *p* < 0.001, η_P_^2^ = 0.329, explaining 57 percent of all variance), while a quadratic contrast, a cubic contrast, and contrasts order 4th to 14th were all insignificant.

#### Feedback expectation

The ANOVA tracking the progression of feedback expectations for the different senders over time showed significant difference in mean expectation ratings between senders (*F*_(2,78)_ = 19.89, *p* < 0.001, η_P_^2^ = 0.338; see Fig. 2B), which was explained by a quadratic (*F*_(1,39)_ = 37.03, *p* < 0.001, η_P_^2^ = 0.487) but not a linear trend (*F*_(1,39)_ = 0.64, *p* = 0.430, η_P_^2^ = 0.016). Post hoc comparisons found that expectation ratings were significantly different between the ‘positive peer’ and the ‘computer’ (*t*_(39)_ = 5.03, *p*_holm_ < 0.001, Cohen’s d = 0.669), as well as between the ‘positive peer’ and the ‘negative peer’ (*t*_(39)_ = 5.81, *p*_holm_ < 0.001, Cohen’s d = 0.772), but did not differ between the ‘negative peer’ and the ‘computer’ overall (*t*_(39)_ = 0.77, *p*_holm_ = 0.441, Cohen’s d = -0.103). There was no statistically significant difference in mean expectation ratings between blocks (*F*_(5,195)*_ = 2.36, *p* = 0.056, η_P_^2^ = 0.057), but there was a significant interaction effect between the factor sender and the factor block (*F*_(10,390)_ = 3.23, *p* < 0.001, η_P_^2^ = 0.077). Follow-up post-hoc comparisons revealed a significant difference between the ‘positive peer’ and the ‘computer’ for the first block (*t*_(39)_ = 3.65, *p*_holm_ = 0.036, Cohen’s d = 0.653), but not between the two peers (*t*_(39)_ = 2.44, *p*_holm_ = 1.00, Cohen’s d = 0435). No significant differences were found between the three senders during the second block (*ts* < 2.26, *ps* = 1.00, Cohen’s ds < 0.404). From the third block onwards, expectation ratings were more positive for the ‘positive peer’ than the ‘computer’ (*ts* > 3.64, *ps* < 0.036, Cohen’s ds > 0.651) and than the ‘negative peer’ (*ts* > 4.44, *ps* < 0.002, Cohen’s ds > 0.793). The ‘negative peer’ did not differ from the ‘computer’ throughout the experiment (*ts* < 2.28, *ps* = 1.00, Cohen’s ds < 0.407). Helmert contrast showed significant linear trends (‘positive peer’, ‘computer’, and ‘negative peer’) for the first (*F*_(1,39)_ = 9.97, *p* = 0.003, η_P_^2^ = 0.204) and second block (*F*_(1,39)_ = 19.81, *p* < 0.001, η_P_^2^ = 0.337), explaining increasing expectation differences during the first two blocks against all subsequent blocks.

A more detailed look at the first block for the first 15 trials showed a significant difference for the factor sender (*F*_(2,78)_ = 9.00, *p* < 0.001, η_P_^2^ = 0.187; see Fig. 2D), again being significantly more positive for the ‘positive peer’ than the ‘computer’ (*t*_(39)_ = 4.16, *p*_holm_ < 0.001), and the ‘negative peer’ (*t*_(39)_ = 2.77, *p*_holm_ = 0.014), the latter two not differing (*t*_(39)_ = 1.39, *p*_holm_ = 0.169). There was a main effect of the trial (*F*_(14,546)*_ = 2.48, *p* = 0.009, η_P_^2^ = 0.060), and an interaction between the sender and trial (*F*_(28,1092)_^*^ = 1.76, *p* = 0.040, η_P_^2^ = 0.043). This interaction in expectations was best explained by a linear contrast (*F*_(1,39)_ = 14.28, *p* < 0.001, η_P_^2^ = 0.268, explaining 45 percent of all variance), while models 7th order (*F*_(1,39)_ = 6.00, *p* = 0.019) and 11th order (*F*_(1,39)_ = 8.66, *p* = 0.005) were also significant.

### ERP results

#### P1

For the P1, analysis for self-view congruence within the peer senders showed no main effects of the sender (*F*_(1,39)_ = 0.33, *p* = 0.571, η_P_^2^ = 0.008) and feedback type (*F*_(1,39)_ = 0.42, *p* = 0.520, η_P_^2^ = 0.011), and no interaction between both (*F*_(1,39)_ = 0.21, *p* = 0.647, η_P_^2^ = 0.005).

Concerning the analyses based on the indicated feedback expectations, no effect of sender (*F*_(1,39)_ = 0.01, *p* = 0.995, η_P_^2^ < 0.001) and feedback type (*F*_(1.43,55.71)_ = 0.22, *p* = 0.729, η_P_^2^ = 0.006), and no interaction was found (*F*_(1.63,63.38)_ = 0.72, *p* = 0.466, η_P_^2^ = 0.018).

Similarly, concerning analyses of trials with a subsequent updating for the peers, there was no main effect for the degree of updating (*F*_(2,78)_ = 0.38, *p* = 0.683, η_P_^2^ = 0.010).

#### N170

For self-view congruence, the N170 showed no main effects of sender (*F*_(1,39)_ = 0.38, *p* = 0.542, η_P_^2^ = 0.010) and feedback type (*F*_(1,39)_ = 0.08, *p* = 0.773, η_P_^2^ = 0.002), and no interaction (*F*_(1,39)_ = 2.07, *p* = 0.158, η_P_^2^ = 0.050).

Concerning the analyses based on the indicated feedback expectations, no effect of sender (*F*_(1,39)_ = 0.94, *p* = 0.935, η_P_^2^ = 0.023) and feedback type (*F*_(2,78)*_ = 1.29, *p* = 0.279, η_P_^2^ = 0.032), and no interaction was found (*F*_(2,78)*_ = 0.01, *p* = 0.988, η_P_^2^ < 0.001).

For trials after which the peer ratings were updated, there was no main effect for the degree of updating (*F*_(2,78)_ = 1.39, *p* = 0.256, η_P_^2^ = 0.034).

#### EPN

**Fig. 3:**
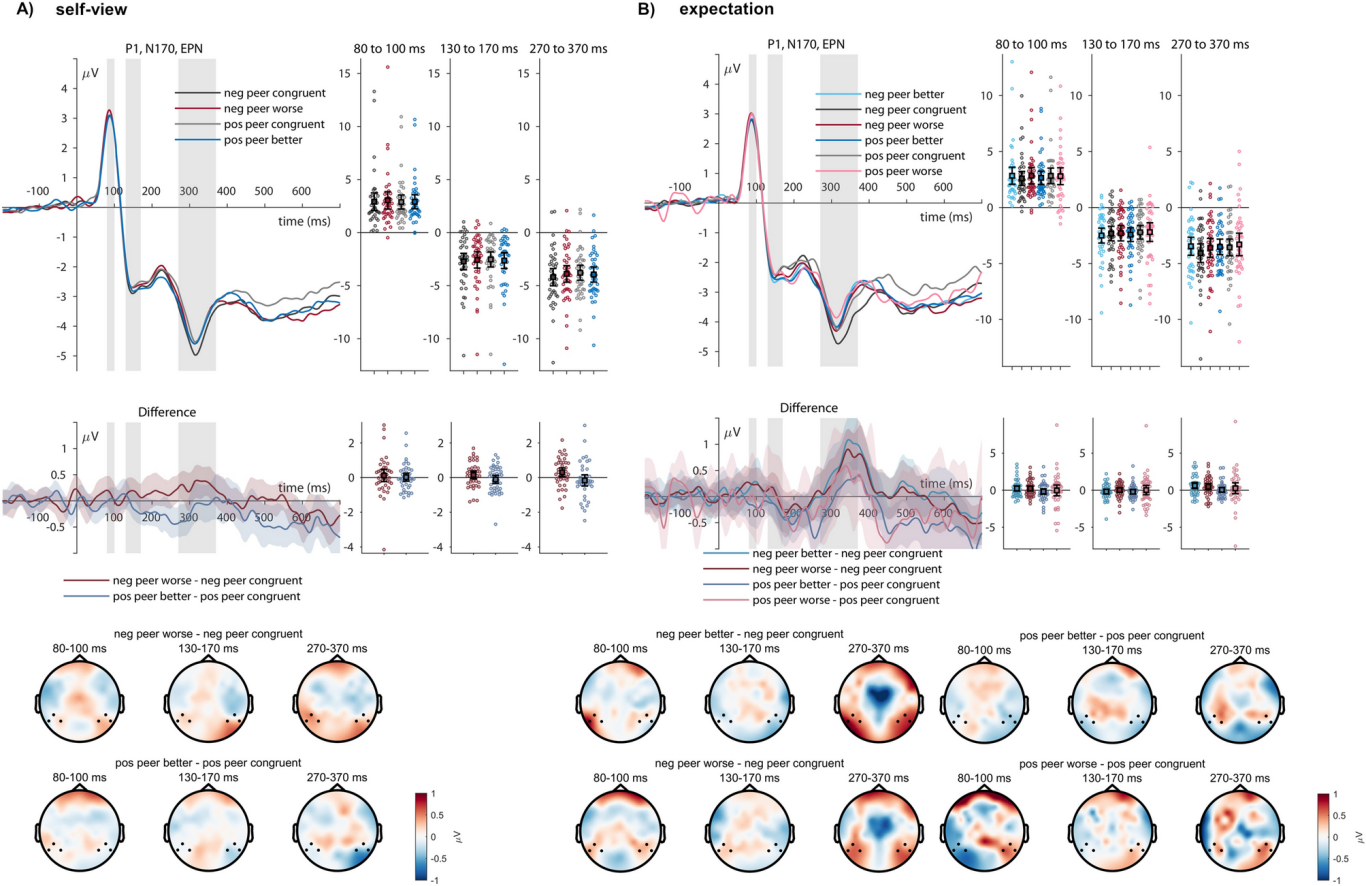
P1, N170, and EPN effects of (**A**) self-view and (**B**) expectation congruence. ERP waveforms show the time course for incongruent (red and blue lines) and congruent feedback (dark and light grey lines) of the ‘positive peer’ and ‘negative peer’. Error bars show 95% confidence intervals. Difference plots below contain 95% bootstrap confidence intervals of intra-individual differences. Scalp topographies depict the amplitude differences between the ‘positive peer’ and the ‘negative peer’ regarding their respective incongruent and congruent feedback.

For the EPN, concerning explorations within self-view congruence of the feedback from the peer senders, no effects of sender (*F*_(1,39)_ = 1.93, *p* = 0.173, η_P_^2^ = 0.047) and feedback type reached significance (*F*_(1,39)_ = 0.37, *p* = 0.549, η_P_^2^ = 0.009), but an interaction was observed (*F*_(1,39)_ = 6.61, *p* = 0.014, η_P_^2^ = 0.145; see Fig. 3A). However, post-hoc tests did not show differences between incongruent and congruent feedback of the ‘positive peer’ (*t*_(39)_ = − 1.08, *p*_holm_ = 0.858, Cohen’s d = − 0.065) and of the ‘negative peer’ (*t*_(39)_ = 2.04, *p*_holm_ = 0.226, Cohen’s d = 0.124).

Concerning the analyses based on the indicated feedback expectations, we observed no effect of sender (*F*_(1,39)_ = 3.90, *p* = 0.056, η_P_^2^ = 0.091), feedback type (*F*_(2,78)*_ = 2.254, *p* = 0.129, η_P_^2^ = 0.054), and no interaction (*F*_(2,78)*_ = 1.31, *p* = 0.273, η_P_^2^ = 0.032).

At the level of the EPN, no effects for the degree of updating were observed (*F*_(2,78)_ = 1.66, *p* = 0.197, η_P_^2^ = 0.041).

#### FRN

**Fig. 4:**
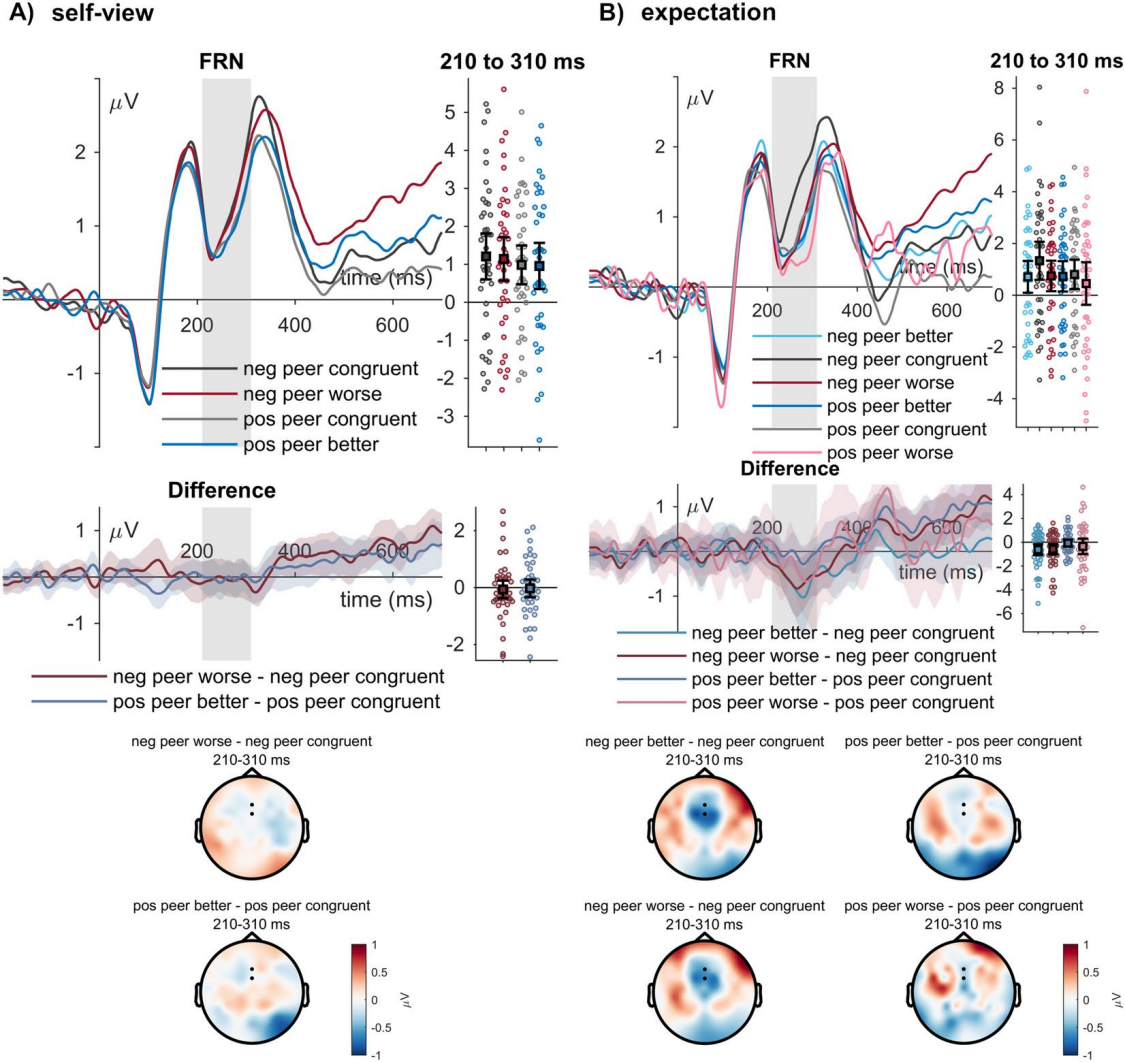
FRN effects of (**A**) self-view and (**B**) expectation congruence. ERP waveforms show the time course for incongruent (red and blue lines) and congruent feedback (dark and light grey lines) of the ‘positive peer’ and ‘negative peer’. Error bars show 95% confidence intervals. Difference plots below contain 95% bootstrap confidence intervals of intra-individual differences. Scalp topographies depict the amplitude differences between the ‘positive peer’ and the ‘negative peer’ regarding their respective incongruent and congruent feedback.

For the FRN, for analyses based on the self-view congruence, explorations within peer senders showed no effects of sender (*F*_(1,39)_ = 3.11, *p* = 0.086, η_P_^2^ = 0.074; see Fig. 4A) and feedback type (*F*_(1,39)_ = 0.17, *p* = 0.682, η_P_^2^ = 0.004), and no interaction (*F*_(1,39)_ = 0.03, *p* = 0.870, η_P_^2^ = 0.001).

Concerning the analyses based on the indicated feedback expectations, we observed an effect of the sender (*F*_(1,39)_ = 7.42, *p* = 0.010, η_P_^2^ = 0.160; see Fig. 4B) and feedback type (*F*_(2,78)*_ = 3.54, *p* = 0.041, η_P_^2^ = 0.083), but no interaction (*F*_(2,78)*_ = 1.43, *p* = 0.246, η_P_^2^ = 0.035). Post-hoc tests showed a relative negativity for the ‘positive’ as compared to the ‘negative peer’, as well as larger negativity for worse feedback than congruent feedback (*t*_(39)_ = -2.61, *p*_holm_ = 0.032, Cohen’s d = − 0.195), but not being significant for better as compared to congruent feedback (*t*_(39)_ = − 0.88, *p*_holm_ = 0.381, Cohen’s d = − 0.066) and between better and worse feedback (*t*_(39)_ = 1.73, *p*_holm_ = 0.174, Cohen’s d = 0.129).

At the level of the FRN, no effects of the degree of updating were observed (*F*_(2,78)_ = 2.28, *p* = 0.109, η_P_^2^ = 0.055).

#### LPP

**Fig. 5:**
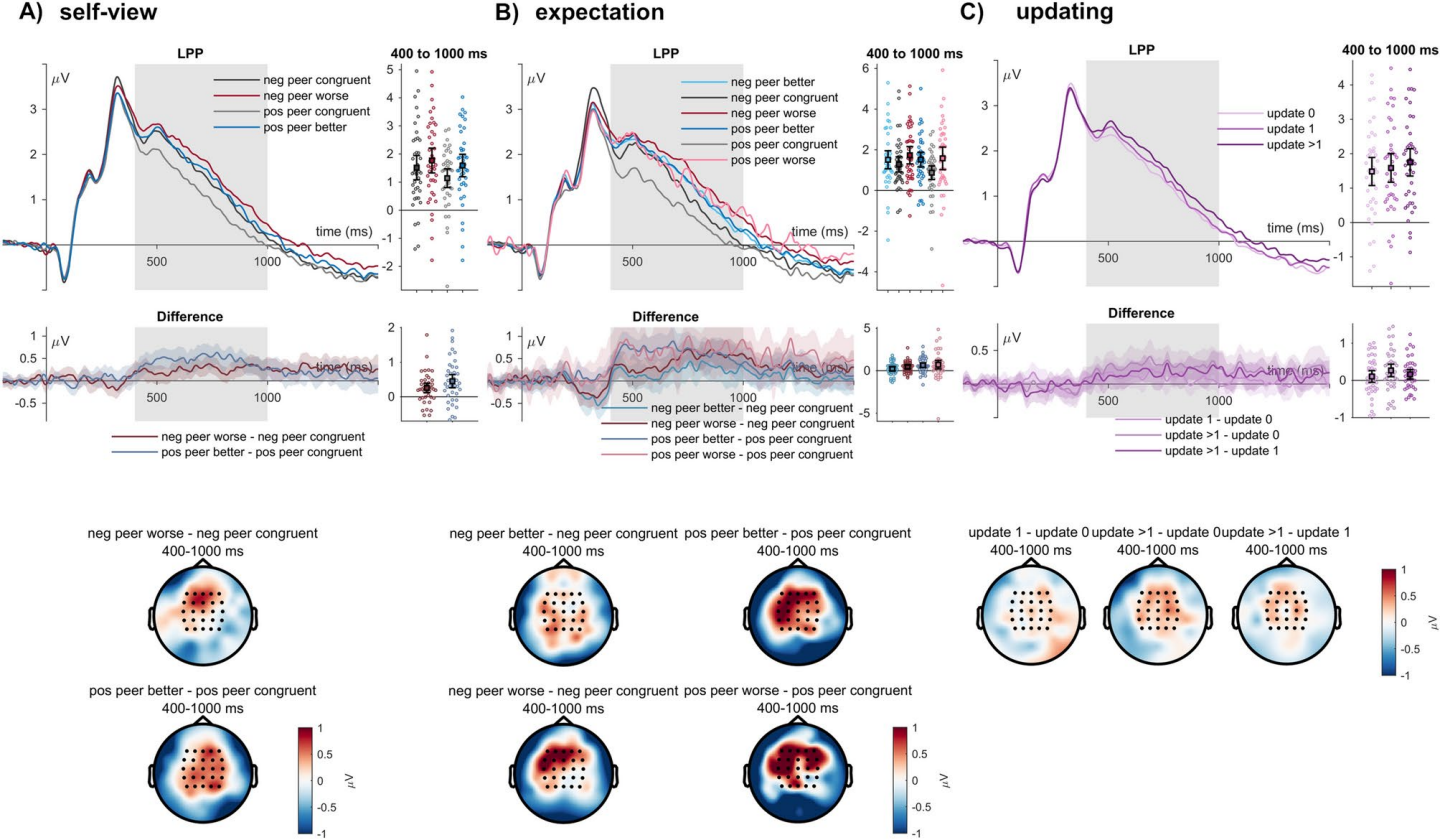
LPP effects of (**A**) self-view and (**B**) expectation congruence, as well as (**C**) subsequent other-rating update. ERP waveforms show the time course for incongruent (red and blue lines) and congruent feedback (dark and light grey lines) of the ‘positive peer’ and ‘negative peer’, and between updating levels (purple lines). Error bars show 95% confidence intervals. Difference plots below contain 95% bootstrap confidence intervals of intra-individual differences. Scalp topographies depict the amplitude differences between the ‘positive peer’ and the ‘negative peer’ regarding their respective incongruent and congruent feedback and between the degree of updating.

For the LPP, congruence with the self-view elicited main effects of sender (*F*_(1,39)_ = 9.04, *p* = 0.005, η_P_^2^ = 0.188; see Fig. 5A) and feedback type (*F*_(1,39)_ = 31.37, *p* < 0.001, η_P_^2^ = 0.446), and no interaction between sender and feedback type (*F*_(1,39)_ = 2.81, *p* = 0.101, η_P_^2^ = 0.067). Post-hoc tests showed a larger positivity for the ‘negative peer’ than the ‘positive peer’ and for incongruent feedback compared to congruent feedback.

Concerning the analyses based on the indicated feedback expectations, we observed effects of the sender (*F*_(1,39)_ = 4.08, *p* = 0.050, η_P_^2^ = 0.095), and of feedback type (*F*_(2,78)_ = 11.64, *p* < 0.001, η_P_^2^ = 0.230; see Fig. 5B), while there was no interaction (*F*_(2,78)*_ = 1.74, *p* = 0.183, η_P_^2^ = 0.043). Post-hoc tests showed a larger positive amplitude for worse feedback than congruent feedback (*t*_(39)_ = 3.49, *p*_holm_ = 0.002, Cohen’s d = 0.316) and better as compared to congruent feedback (*t*_(39)_ = 4.63, *p*_holm_ < 0.001, Cohen’s d = 0.419). There were no differences between better and worse feedback than expected (*t*_(39)_ = 1.14, *p*_holm_ = 0.260, Cohen’s d = 0.103).

Concerning the updating analyses, the LPP revealed a significant main effect for the degree of updating (*F*_(2,78)_ = 5.79, *p* = 0.005, η_P_^2^ = 0.129, see Fig. 5C). A larger positivity was observed for feedback trials, after which participants changed the evaluation by two or more points compared to trials during which participants maintained their rating (*t*_(39)_ = 3.38, *p*_holm_ = 0.003, Cohen’s d = 0.191). The contrast between updating1 and updating 0 was not significant (*t*_(39)_ = 1.32, *p*_holm_ = 0.190, Cohen’s d = 0.075), similar between updating > 1 and updating 1 (*t*_(39)_ = 2.05, *p*_holm_ = 0.087, Cohen’s d = 0.116).

## Discussion

Social feedback is everywhere^78^. Positive feedback enhances self-efficiency, flow, and performance^79^, even including motor development in children^80^. Positive feedback can aid cognitive and social development, foster a positive self-concept, support mental health, and enhance performance in both educational and professional settings, while negative social feedback also has manifold detrimental effects. This study tested how real social interaction and the following experimentally manipulated social evaluative feedback affect ERPs to feedback and behavioral responses. This included the change in feedback expectations and sender-related trait ratings. We observed the predicted general change in feedback expectations and the ratings of the senders following their respective positive or negative feedback. Interestingly, trait ratings for the peers changed rapidly following positive or negative feedback. Concerning ERPs, we tested for modulations based on congruence both with the self-view and with the participants’ feedback expectations. We observed no differences between the peer senders and feedback congruence on early ERPs, while expectation congruence modulated the FRN component, with a larger negativity for feedback being worse than expected. During the LPP, we observed that incongruence, both with the self-view and feedback expectation, increased LPP amplitudes. Finally, the degree of updating traits of the peers was related to increased LPP amplitudes.

We tested for the first time how social evaluative feedback changes the expectation and evaluation of traits associated with the sender. Most importantly, concerning other-related updates, some accounts speak for a stronger impact of positive information, which is typically related to higher self-related integration of the information^81^. We observed clear effects of feedback sender valence that emerged already in the first feedback block. We find that the positive and negative peer are rated over time more positively and negatively, according to their feedback behavior. While trait-related feedback has not been examined in this aspect, similar effects were reported observed in studies on social evaluative feedback, with a higher-rated pleasantness or mood after positive feedback^82,83^, or a rated higher liking of co-players in the Island Getaway task when receiving positive feedback^10,84^. Other correlational approaches show positively biased memory traces for positive feedback or for supposed senders of positive feedback^14,85,86^. We find that trait ratings were changing both for the positive and negative sender. Here, a recent publication suggested engagement vs. disengagement in response to negative feedback to be functional depending on behavioral adaptation^87^. Behavioral adaptation in our study could be framed by the re-rating of the sender and showing reciprocity by increasing one’s ratings in a positive direction by positive feedback^10^ or decreasing ratings after negative feedback^13–16^. It would be interesting to test whether negative effects (e.g., on mood or self-esteem) of negative social evaluative feedback are attenuated by the possibility of reciprocally decreasing own ratings of the feedback-sender, as in our study. Negative social evaluative feedback is related to negative affect^88^, decreases in mood and self-esteem^89,90^, and psychiatric disorders, such as depression and anxiety^91,92^.

Furthermore, we tested for changes in feedback expectations. Here, initial feedback expectations were moderately positive concerning the used scale values (rated positivity approximately 6.5 of 9), in line with findings for healthy participants in other research paradigms^17,19,20^. Concerning the change in feedback expectations, we found the ‘positive peer’ differentiation during the first block compared to the ‘computer’ but not against the other peer. More positive feedback was expected for the ‘positive peer’ from the third block onwards. This is a somewhat slower evolvement than for the other-rating updating processes. Feedback has been suggested to fulfill the psychological need to belong to a social group crucial to survival in the past^1^. Humans seek positive evaluative feedback, as it increases positive affect^93^, self-efficacy^94^, resilience^95^, and feelings of competence and autonomy^96^. We cannot outrule the fact that changes in feedback expectations might have been influenced by the task that requires explicit attention to update the other person. Some research has shown that self- vs. other-related learning seems to differ, at least in social evaluative tasks where socially anxious participants showed distorted self-related learning but no deficits in learning about other persons’ behavior^91^.

Concerning the corresponding ERPs, no effects of congruence with the self-view or feedback expectation were observed on early ERPs (P1, N170), but during mid-latency time windows (EPN, FRN). EPN effects for self-view incongruence differed between the two peer senders, while post-hoc comparisons were insignificant. In contrast, we observed that the FRN was not affected by the self-view congruence but by incongruence with the participants’ feedback expectations. Here, we found an increase in feedback that was worse than expected, which some social evaluative feedback studies have also shown^8^. However, one should interpret this selectivity for worse feedback cautiously since both unexpected worse and better feedback within the negative peer elicited similar increases in the FRN amplitude, as some views related the FRN more to the expectedness of outcomes^57,97^. Furthermore, the effects of worse feedback from the positive peer were based on the least trial numbers (see Table 2). There was no significant interaction, while descriptively, FRN differences between unexpected feedback conditions differed between the peers. Specifically, the rather frequent ‘better than expected’ feedback from the ‘positive peer’ elicited no FRN modulation. It may be a dissociation between the indicated and the actual expectation, where participants might have indeed already expected more positive feedback. During the late processing stages, participants generally showed larger LPP amplitudes to incongruent feedback. This was observed both for the self-view and the feedback expectations. These analyses within the two peer senders showed that incongruent (i.e., positive or negative) feedback elicited enhanced processing that may be related to the integration of the feedback or updating-related processes^3^.

Additionally, we tested ERP-behavioral relationships. Here, trials in which participants substantially changed their ratings correlated with the LPP. We observed an increased LPP amplitude for larger changes of the peer senders (i.e., trials with a difference of more than one point). This is interesting, as, so far, a relationship between ERP responses to social evaluative feedback and behaviour has rarely been made^84,85^. One study correlated ERPs during social evaluative feedback and subsequent memory performance measures, showing a moderate relationship between slow wave amplitudes and a more liberal memory bias for previous positive social evaluative feedback^85^. These reports, together with our findings, align with the proposed mechanism of the late ERP responses, where elaborate processing, extracting evaluation meaning, and updating processes are suggested to take place^27^. While the observed relationship between LPP amplitudes during feedback and following rating patterns is only correlational, it seems an interesting starting point for future studies. Finally, we confirmed that the attributed social evaluative sender relevance increases early (P1, N170), intermediate (EPN), and late (LPP) ERPs (see Supplementary Section 1 and Supplementary Figs. S1 and S2) ^8,29,53,54^. A caveat of interpreting the early effects here is that our study provided participants with important context information in advance. The information per mini-block, in which the sender would provide feedback, enabled the preparation for feedback within the more relevant condition. Early effects of sender might, therefore, represent differences in anticipation of feedback^29,55^, while revealing sender and feedback information also leads to higher complexity of information integration that may delay modulations^29,53–55^.

### Constraints of generality and outlook

Our design is based on real-life interactions between the participant and two unknown peers, with some limitations in the current design, including the real interaction scenario. We realized a scripted interview with no restrictions on how people present themselves, allowing natural interaction between all partners and lacking precise control over the impressions participants made and received. This ensured high ecological validity, and no participant doubted its veracity. Secondly, the short time delay between the other evaluation and re-evaluation prevented memory distortions and prevented the peers from chatting with each other after the first sessions and exchanging ratings. It might have caused a bias for self-coherence of the ratings. The degree of other updates might be underestimated. This is particularly important in relation to predominantly positive feedback from a sender without the direct equivalent of negative feedback, as overly positive feedback might influence the degree of positive self-disclosure in healthy individuals^98^. On the other hand, as recent research has shown, expressed opinions tend to be more extreme in cases when negative feedback is not submitted^99^. Furthermore, understanding changes in expectations on the one hand and changes in the other-views and self-view would be highly interesting to study in different patient samples to understand the origin and consequences of pathological processes.

## Conclusion

The current study reports the effects of feedback behavior on feedback expectations and other-view updates, with rapid changes in the other-view and also differences in feedback expectations for the two peers. Other-view updating and feedback ratings were more positive for the positive sender and negative for the negative sender. We observed the earliest ERP modulations for incongruent feedback with the self-view (EPN) and the feedback expectation (FRN) during the mid-latency stages, while both types of incongruence increased LPP amplitudes. Possibly, this relates to elaborate processing and updating mechanisms, as larger changes in the other-view were also related to larger LPP amplitudes. Finally, relevant evaluative feedback (social vs. non-social source) increased the whole time course of feedback processing.

## Supplementary Information

Supplementary Information.

## Data Availability

The data that support the findings of this study have been deposited in the Open Science Framework (https://osf.io/q9yue/). All EEG and behavioural data, participant information, and experimental design information are available in the respective repository.
