# Supplementary material for: How positive and negative feedback following real interactions changes subsequent sender ratings
Source: Sci Rep. 2025 Mar 3;15:7470. doi: 10.1038/s41598-025-91750-1 (PMC11876303; doi:10.1038/s41598-025-91750-1)
Supplement: Supplementary file 1 — Supplementary Information. [file 41598_2025_91750_MOESM1_ESM.docx]

**How positive and negative feedback following real interactions changes subsequent sender ratings**

**SUPPLEMENTARY MATERIALS**

Antje Peters^1,2*┼^, Jendrik Witte^1┼^, Hanne Helming^1^, Robert Moeck^1^, Thomas Straube^1,2^, and Sebastian Schindler^1,2^*

^1^Instutite for Medical Psychology and Systems Neuroscience, University of Münster, Germany

^2^ Otto Creutzfeldt Center for Cognitive and Behavioral Neuroscience, University of Muenster

* Corresponding author

^┼^Co-first authors

**Correspondence address**

Institute of Medical Psychology and Systems Neuroscience

University of Münster

Von-Esmarch-Str. 52, D-48149 Münster, Germany

e-mail: [sebastian.schindler@ukmuenster.de](mailto:sebastian.schindler@ukmuenster.de)

1. **ERP analyses comparing peer and computer feedback.**

For ERP analyses, we performed repeated Measures ANOVAs of the sender across all three sender conditions (three levels: 'positive peer', 'negative peer', and computer).

**P1**

## For the P1, the effect of sender reached significance (*F*_(2,78)_ = 5.69, *p =* .005, η_P_² = .127; see Supplementary Figure S1). There was a significantly increased P1 for the 'positive peer' (*t*_(39)_ = 2.72, *p*_holm_ = .016, Cohen's d = 0.270) and the 'negative peer' compared to the computer feedback (*t*_(39)_ = 3.09, *p*_holm_ = .008, Cohen's d = 0.306), while the peer conditions not differing from each other (*t*_(39)_ = -0.368, *p*_holm_ = .714, Cohen's d = -0.036).

**N170**

## Likewise, the N170 was affected by the feedback sender (*F*_(2,78)_ = 34.69, *p <* .001, η_P_² = .127), with increased negativity for the 'positive peer' (*t*_(39)_ = -6.97, *p*_holm_ < .001, Cohen's d = -0.512) and the 'negative peer' compared to the computer feedback (*t*_(39)_ = -7.37, *p*_holm_ < .001, Cohen's d = -0.541). Again, the peer conditions did not differ from each other (*t*_(39)_ = 0.40, *p*_holm_ = .692, Cohen's d = 0.029).

**EPN**

For the EPN, as affected by the feedback sender (*F*_(2,78)_ = 52.81, *p <* .001, η_P_² = .575), with increased negativity for the 'positive peer' (*t*_(39)_ = -8.54, *p*_holm_ < .001, Cohen's d = -1.027) and the 'negative peer' compared to the computer feedback (*t*_(39)_ = -9.22, *p*_holm_ < .001, Cohen's d = -1.109). Again, the peer conditions did not differ from each other (*t*_(39)_ = 0.68, *p*_holm_ = .496, Cohen's d = 0.082).

##
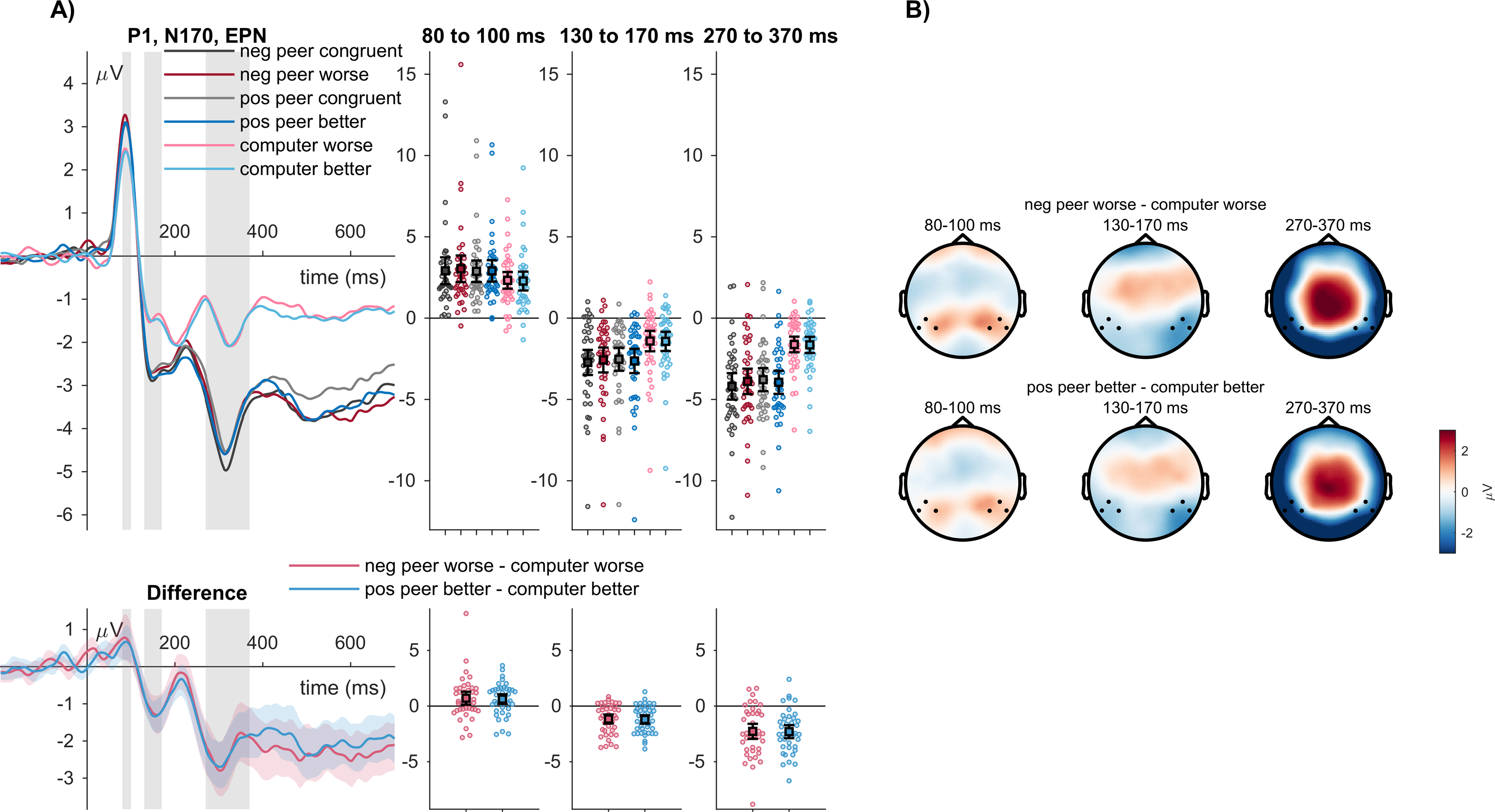
Supplementary Figure S1. P1, N170, and EPN effects of the sender. A) ERP waveforms show the time course for incongruent and congruent feedback of the 'positive peer' (blue lines) and 'negative peer' (red lines), as well as the positive and negative computer feedback (bright blue and red lines, respectively). Error bars show 95% confidence intervals. Difference plots below contain 95% bootstrap confidence intervals of intra-individual differences. B) Scalp topographies depict the amplitude differences between the positive (pos) and negative (neg) peers for their incongruent feedback against the respective computer feedback.

## LPP

For the LPP, the effect of the sender was again significant (*F*_(2,78)_ = 39.92, *p <* .001, η_P_² = .506; see Supplementary Figure S2). A larger positivity was observed for the negativity for the 'positive peer' (*t*_(39)_ = 6.62, *p*_holm_ < .001, Cohen's d = 0.814) and the 'negative peer' compared to the computer feedback (*t*_(39)_ = 8.51, *p*_holm_ < .001, Cohen's d = 1.045). The peer conditions did not differ significantly from each other (*t*_(39)_ = 1.89, *p*_holm_ = .063, Cohen's d = 0.232).

**
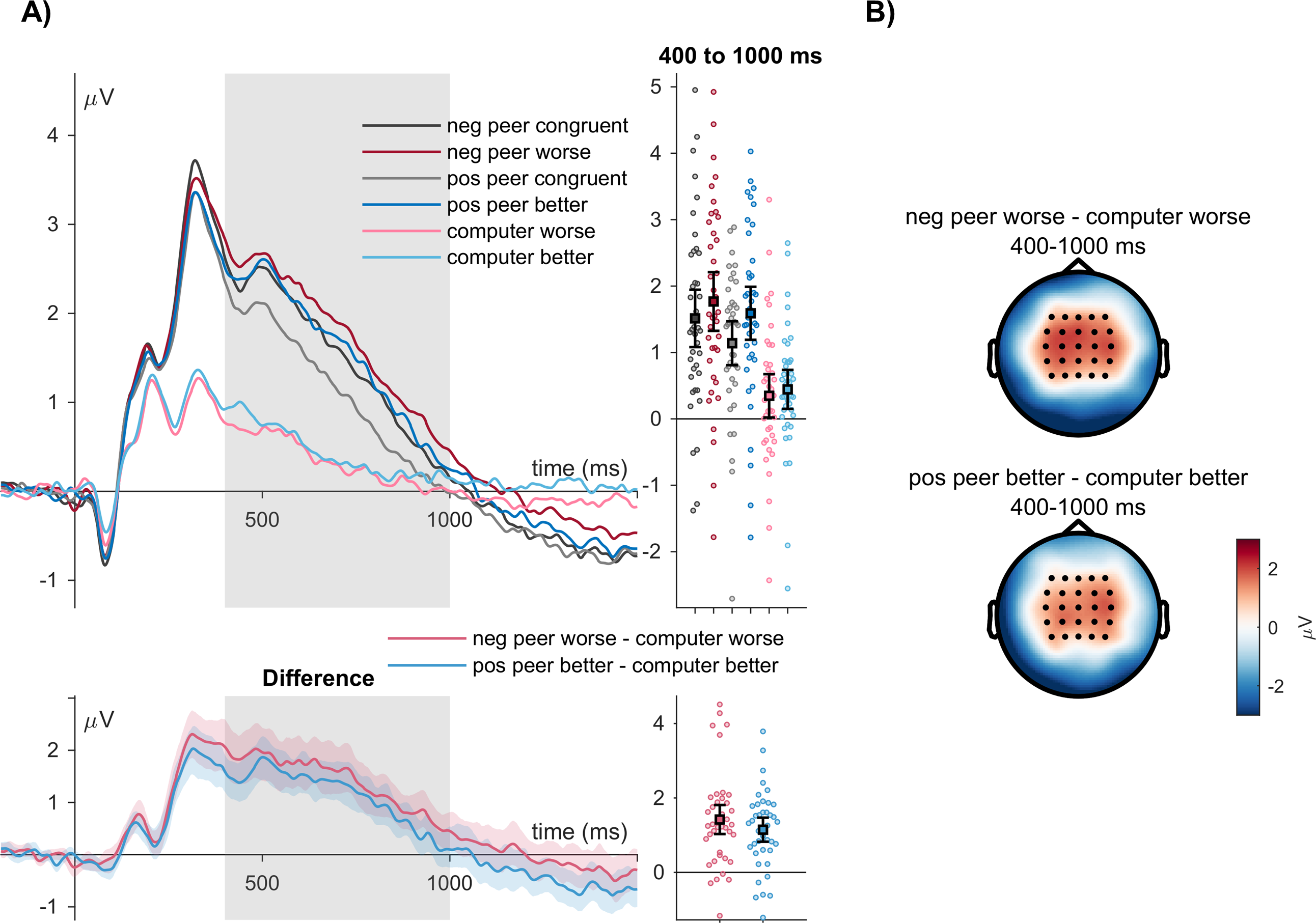
Supplementary Figure S2. LPP effects of the sender. A)** ERP waveforms show the time course for incongruent and congruent feedback of the 'positive peer' (blue lines) and 'negative peer' (red lines), as well as the positive and negative computer feedback (bright blue and red lines, respectively). Error bars show 95% confidence intervals. Difference plots below contain 95% bootstrap confidence intervals of intra-individual differences. **B)** Scalp topographies depict the amplitude differences between the positive (pos) and negative (neg) peers for their incongruent feedback against the respective computer feedback.
